# Transcriptomes of *Saussurea* (Asteraceae) Provide Insights into High-Altitude Adaptation

**DOI:** 10.3390/plants10081715

**Published:** 2021-08-20

**Authors:** Xu Zhang, Yanxia Sun, Jacob B. Landis, Jun Shen, Huajie Zhang, Tianhui Kuang, Wenguang Sun, Jiao Sun, Bashir B. Tiamiyu, Tao Deng, Hang Sun, Hengchang Wang

**Affiliations:** 1 CAS Key Laboratory of Plant Germplasm Enhancement and Specialty Agriculture, Wuhan Botanical Garden, Chinese Academy of Sciences, Wuhan 430074, China; zhangxu173@mails.ucas.ac.cn (X.Z.); sunyanxia@wbgcas.cn (Y.S.); zhanghuajie@wbgcas.cn (H.Z.); sunjiao19@mails.ucas.ac.cn (J.S.); tiamiyu.bb@unilorin.edu.ng (B.B.T.); 2 Center of Conservation Biology, Core Botanical Gardens, Chinese Academy of Sciences, Wuhan 430074, China; 3 University of Chinese Academy of Sciences, Beijing 100049, China; 4 Section of Plant Biology and the L.H. Bailey Hortorium, School of Integrative Plant Science, Cornell University, Ithaca, NY 14850, USA; jbl256@cornell.edu; 5 BTI Computational Biology Center, Boyce Thompson Institute, Ithaca, NY 14853, USA; 6 State Key Laboratory of Rice Biology, Zhejiang Provincial Key Laboratory of Crop Genetic Resources, Institute of Crop Science, College of Agriculture and Biotechnology, Zhejiang University, Hangzhou 310058, China; 12016030@zju.edu.cn; 7 Key Laboratory for Plant Diversity and Biogeography of East Asia, Kunming Institute of Botany, Chinese Academy of Sciences, Kunming 650201, China; kuangtianhui19@mails.ucas.ac.cn (T.K.); sunwenguang@mail.kib.ac.cn (W.S.); dengtao@mail.kib.ac.cn (T.D.); 8 School of Life Science, Yunnan Normal University, Yunnan 650500, China

**Keywords:** high-altitude adaptation, *Saussurea*, transcriptomes, positive selection, extreme environments, lineage-specific genes

## Abstract

Understanding how species adapt to extreme environments is an extension of the main goals of evolutionary biology. While alpine plants are an ideal system for investigating the genetic basis of high-altitude adaptation, genomic resources in these species are still limited. In the present study, we generated reference-level transcriptomic data of five *Saussurea* species through high-throughput sequencing and *de novo* assembly. Three of them are located in the highland of the Qinghai-Tibet Plateau (QTP), and the other two are close relatives distributed in the lowland. A series of comparative and evolutionary genomics analyses were conducted to explore the genetic signatures of adaptive evolution to high-altitude environments. Estimation of divergence time using single-copy orthologs revealed that *Saussurea* species diversified during the Miocene, a period with extensive tectonic movement and climatic fluctuation on the QTP. We characterized gene families specific to the alpine species, including genes involved in oxidoreductase activity, pectin catabolic process, lipid transport, and polysaccharide metabolic process, which may play important roles in defense of hypoxia and freezing temperatures of the QTP. Furthermore, in a phylogenetic context with the branch model, we identified hundreds of genes with signatures of positive selection. These genes are involved in DNA repair, membrane transport, response to UV-B and hypoxia, and reproductive processes, as well as some metabolic processes associated with nutrient intake, potentially responsible for *Saussurea* adaptation to the harsh environments of high altitude. Overall, our study provides valuable genomic resources for alpine species and gained helpful insights into the genomic basis of plants adapting to extreme environments.

## 1. Introduction

Understanding the genetic basis of species adapting to high altitude is a long-standing theme of evolutionary biology [1,2,3,4,5]. Species inhabiting high-altitude environments have to face a variety of abiotic stresses, such as reduced oxygen availability, rapid fluctuations in temperature, and high ultraviolet (UV) radiation [6,7,8]. These stresses present strong selective pressure and have driven striking phenotypic and genetic adaptations [9,10,11,12]. Advances in high-throughput sequencing, especially RNA sequencing (RNA-Seq), enable the collection of gene sequences for non-model species [13], opening up a new era for comparative evolutionary studies on genetic adaptations [14,15,16].

The Qinghai-Tibet Plateau (QTP) is the highest (average elevation above 4000 m) and the largest (ca. 2.5 million km^2^) plateau in the world [17,18], representing an ideal natural laboratory for investigating ecological adaptation in high-altitude environments. The uplift of the QTP creates profound climatic and environmental diversity, making this area one of the world’s richest temperate floras [19,20,21]. While several genome-wide studies on adaptive evolution on the QTP have been conducted, including humans [2,9], plants [4,5,6], vertebrates [11], and bacteria [22], our knowledge of genetic adaptation to high-altitude is far from adequate, due to empirical studies showing that different organisms adapt to high altitudes via multiple genetic routes [11]. Especially in the alpine screes of the QTP (usually elevation above 4500 m), characterized by freezing temperatures, high winds, strong UV rays, and poor soil quality, plants have evolved extraordinarily diverse traits for adaptation, such as leafy bracts, woolly trichomes and cushion plants [23,24,25]. Despite increased understanding of the ecological benefits of these adaptive traits [26,27], the genetic mechanisms underlying high-altitude adaptation are still poorly studied.

*Saussurea* DC. is one of the most species-rich genera in Asteraceae [28], comprising approximately 400 species, traditionally recognized as four subgenera, distributed in Asia, Europe, and North America, with the highest diversity in the QTP [28,29]. Species in *Saussurea* are present in all possible terrestrial habitats, including steppes, moist forests, cold and dry alpine meadows, and scree slopes above 5000 m, demonstrating the capability to be highly adaptive [28,29,30,31]. Previous investigations indicated that *Saussurea* originated in the middle Miocene, and the uplift of the QTP promoted the rapid diversification of this alpine lineage [30,32]. In addition, *Saussurea* exhibits extraordinary morphological diversity, such as species with woolly trichomes, so-called ‘snow rabbits’, in *S*. subg. *Eriocoryne*, and species with leafy bracts, so-called ‘snow lotuses’, in *S*. subg. *Amphilaena* [28,29]. A recent study revealed that these attractive traits of *Saussurea* are the result of convergent adaptation to high-altitude environments [31] and are prevalent among species inhabiting alpine screes of the QTP [26], making the genetic basis behind ecological adaptation more enigmatic. 

In the present study, we sampled three *Saussurea* species from alpine screes of the QTP (*S. pachyneura* Franch., *S. salwinensis* J. Anthony, and *S. velutina* W.W. Smith) as well as two lowland relatives (*S. amara* (L.) DC. and *S. amurensis* Turcz. ex DC.) to generate reference-level transcriptome sequences using RNA-seq and *de novo* assembly. By conducting comparative and evolutionary genomics analyses, including the detection of lineage-specific gene families and genes under positive selection on different branches, we aim to gain insights into the genetic basis of adaptive evolution to high-altitude environments at the genomic level.

## 2. Results

### 2.1. De novo Assembly and Annotation of Unigenes

**Figure 1:**
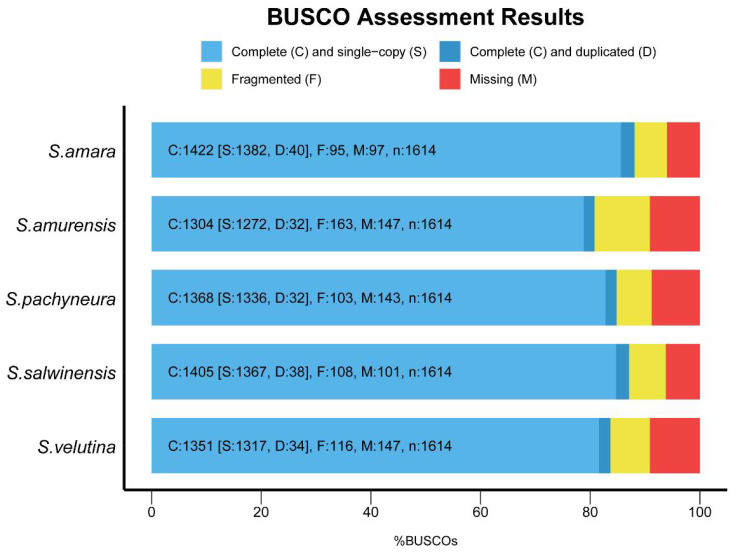
BUSCO assessment results for the five assembled transcriptomes of *Saussurea*. Bar charts produced with the BUSCO plotting tool show proportions classified as complete (C, blues), complete single-copy (S, light blue), complete duplicated (D, dark blue), fragmented (F, yellow), and missing (M, red).

We generated 80.0–92.1 million reads per species, yielding 12.0–13.8 Giga-base (Gb) of RNA-seq data per species. After quality control, 79.8–92.0 million reads per species remained, consisting of 11.7–13.6 Gb clean data per species (Supplementary Table S2). *De novo* assembly yielded 176,265–270,778 transcripts with 151.5–237.3 million base and 91,980–147,303 unigenes with 65.9–87.5 million base, respectively. The median length of transcripts was 417 bp–611 bp (mean length: 646.45 bp–967.28 bp), and the median length of unigenes was 353 bp–427 bp (mean length: 574.69 bp–763.58 bp), with contig N50s ranging from 899 bp to 1562 bp for transcripts and from 787 bp to 1334 bp for unigenes (Table 1). BUSCO results showed that the completeness of *de novo* assembly reached 88.1%, 80.8%, 84.8%, 87.1%, and 83.7% for *S. amara, S. amurensis, S. pachyneura*, *S. salwinensis,* and *S. velutina,* respectively (Figure 1), suggesting high-level quality.

**Figure 2:**
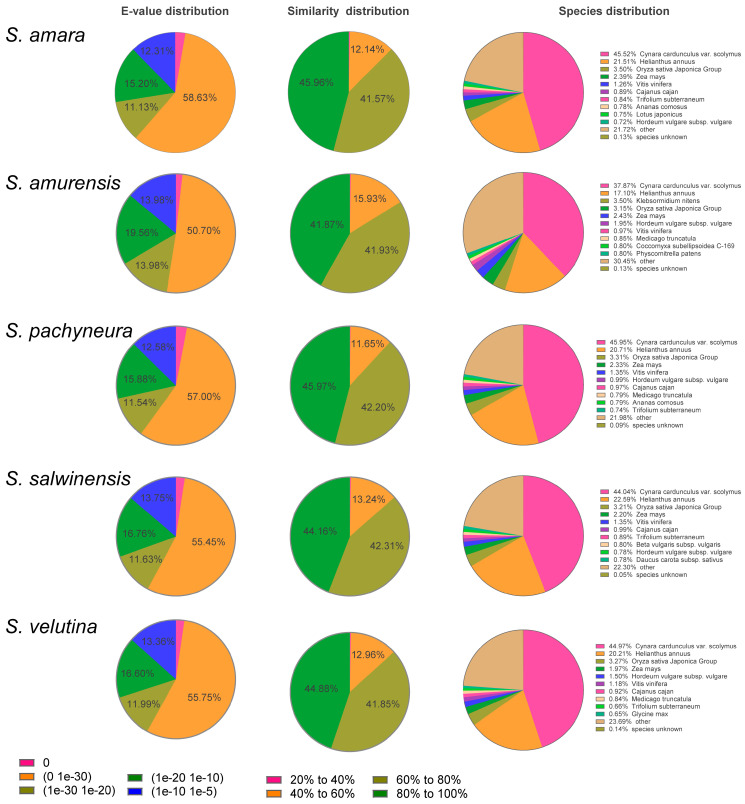
The proportion of the unigenes of *S. amara, S. amurensis, S. pachyneura, S. salwinensis*, and *S. velutina* annotated to NCBI non-redundant protein database. For species distributions, the top 10 matched species are provided.

TransDecoder identified 16,851, 28,182, 15,906, 19,979, and 19,978 protein-encoding regions for *S. amara, S. amurensis, S. pachyneura, S. salwinensis,* and *S. velutina*, respectively, consisting of 16.53–19.13% of unigenes for each species (Table 2). All the unigenes were annotated based on similarity to the public NR, Swiss-Prot, Pfam COG, KEGG, and GO databases. NR had the highest proportion of successful annotations, while KEGG had the lowest proportion (Table 2). The two top hits for the three species in the NR database are *Cynara cardunculus* var. *scolymus* and *Helianthus annuus* (Figure 2). Three functional categories: cellular component, molecular function, and biological process were classified based on the GO terms of all unigenes (Supplementary Figures S1–S5).

### 2.2. Estimation of Divergence Time 

**Figure 3:**
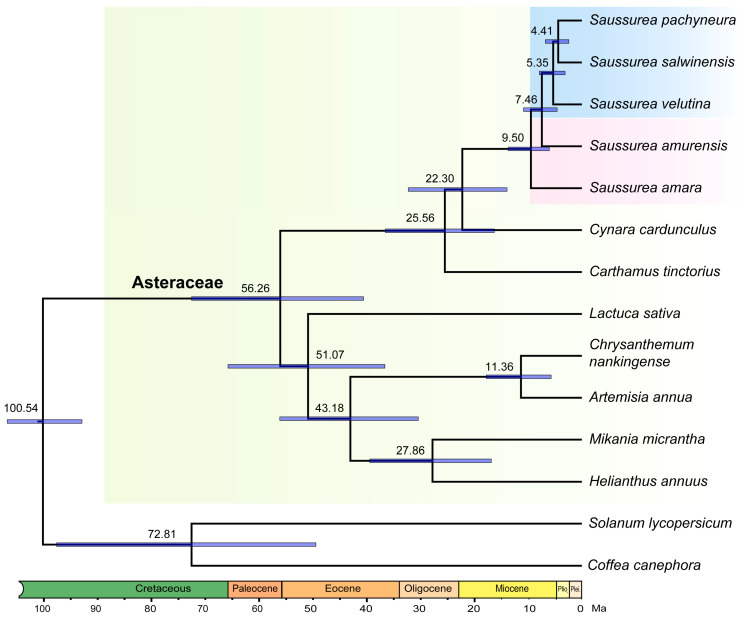
Estimated time tree of *Saussurea* based on 1136 single-copy orthologous gene sequences. Mean node ages are provided, and node bars provided are 95% HPD intervals for mean node ages. The three alpine species and two lowland relatives are in the blue and pink background, respectively.

A total of 1136 single-copy orthologous genes were used for phylogenetic reconstruction. The resulting phylogeny (Figure 3) is consistent with known phylogenetic relationships within Asteraceae. The topology shows that the two lowland species form a clade and together are sisters to the three alpine species. Among the alpine species, *S. velutina* is sister to *S. pachyneura* and *S. salwinensis* (Figure 3). According to the MCMCTree analysis, the crown group of *Saussurea* diverged at ca. 9.50 million years ago (Mya) (95% highest posterior density (HPD): 6.08–13.76 Mya), and all *Saussurea* species are estimated to have originated in the late Miocene to the early Pliocene (4.41–9.50 Mya). The divergence time between *Saussurea* and *Cynara* is estimated to be ca. 22.30 Mya (95% HPD: 13.95–32.37 Mya). The divergence time of Asteraceae species is estimated to diverge at ca. 56.26 Mya (95% HPD: 40.70–72.81 Mya) (Figure 3). 

### 2.3. Evolutionary Analysis

**Figure 4:**
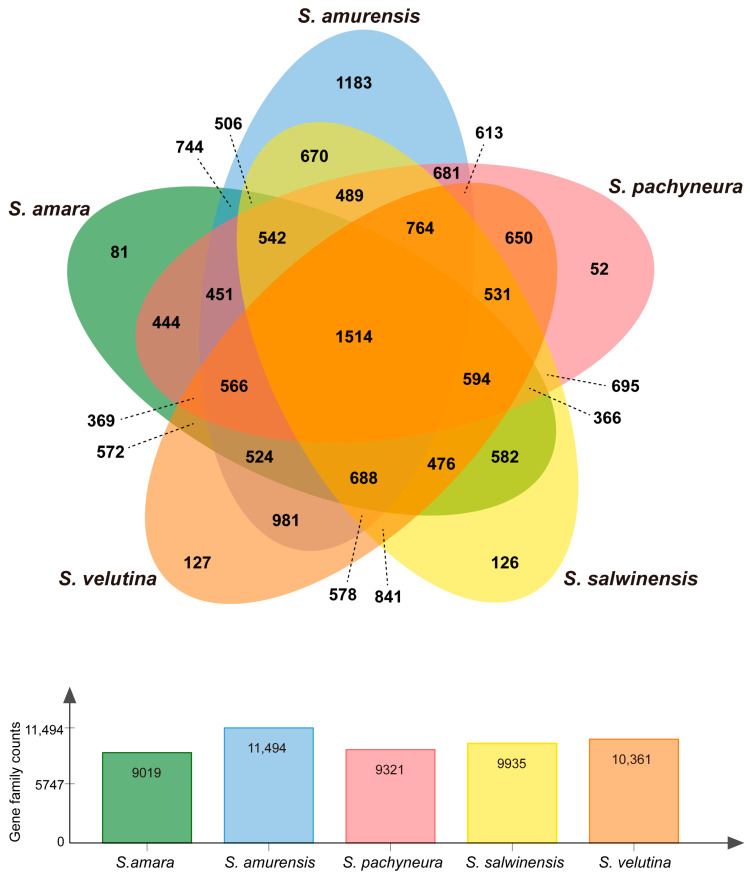
Gene family comparison among five *Saussurea* species. Numbers on the graph represent the count of gene families. Gene Ontology (GO) enrichment for lineage-specific gene families of three alpine species (*S. pachyneura, S. salwinensis,* and *S. velutina*) are provided in Table 3.

OrthoVenn2 identified 17,380 orthologous clusters (containing at least two species) among five *Saussurea* species, which includes 1514 core orthologs (shared by all five species) (Figure 4). The number of lineage-specific gene families for three alpine species, *S. pachyneura, S. salwinensis,* and *S. velutina*, are 52, 126, and 127, respectively. GO enrichment was then conducted on the lineage-specific gene families by annotating them with UniProt. In total, 14 significant GO terms (*p*-value < 0.05) are enriched (three for *S. pachyneura*, five for *S. salwinensis*, and six for *S. velutina*) (Table 3). These GO terms included genes involved in oxidoreductase activity (GO: 0016705; GO:0016491), pectin catabolic process (GO:0045490), lipid transport (GO:0006869), and polysaccharide metabolic process (GO:0005976), which may play important roles in defense of hypoxia and freezing temperatures of the QTP. 

### 2.4. Genes under Positive Selection

**Figure 5:**
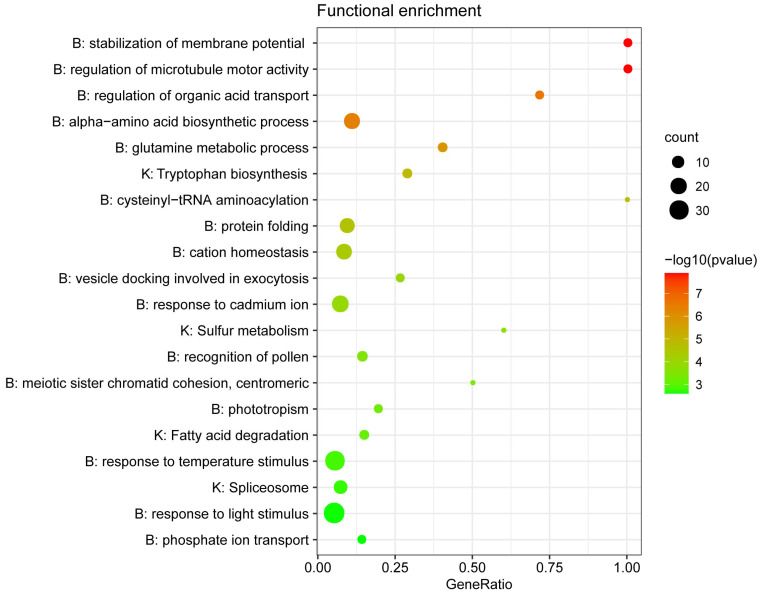
Functional enrichment analysis of genes under positive selection. The top 20 clusters with their representative enriched terms (one per cluster) of GO biological processes (B) and KEGG pathways (K) are shown.

To detect genes that might contribute to the high-altitude adaption of the alpine species (*S. pachyneura*, *S. salwinensis*, and *S. velutina*), we used the branch model to screen positively selected genes (PSGs) by estimating the d*N*/d*S* ratio on each branch of the alpine species using orthologous genes of five *Saussurea* species as input. In total, 250 genes across all three high-altitude species displayed signs of positive selection, of which 215 genes were successfully annotated with at least one homologous gene matched to *A. thaliana* (Supplementary Table S3). Functional enrichment analysis revealed 136 GO processes and 24 KEGG pathways significantly enriched (Supplementary Table S4). The top 20 clusters with their representative-enriched terms (one per cluster) are shown in Figure 5. Numerous PSGs show significant enrichment in biological functions associated with harsh environment defense, such as stabilization of membrane potential (e.g., GO:0030322, GO:0042391), regulation of transmembrane transport (e.g., GO:0098662, GO:2000576, GO:0032890), reproductive process (e.g., GO:0048544, GO:0051754), response to temperature (GO:0009266) and light stimulus (GO:0009416), and are also involved in important metabolic processes associated with nutrient or energy intake, such as glutamine metabolic process (GO:0006541, GO:0009064), sulfur metabolism (KEGG: ko00920, GO:0000096) and tryptophan biosynthesis (KEGG: M00169, GO:0000162). In addition to the significantly enriched pathways, many PSGs are annotated to homolog proteins of *A. thaliana* that are important for species adaptation. For example, RAD54, RUP2, TT5, and AT5G35735 are involved in DNA repair, response to UV-B, flavonoid biosynthesis, and cellular response to hypoxia.

## 3. Discussion

Despite *de novo* assembly due to the lack of whole-genome sequence, transcriptomes of *Saussurea* generated in this study are of high quality. BUSCO results showed a minimum of 81% completeness (up to 88%) for the five transcriptomes (Figure 1), providing a strong foundation for our following comparative transcriptomic analyses. Using single-copy orthologs identified in genomic sequences, we added transcriptomic evidence of the divergence history of *Saussurea.* Our dated time tree showed the divergence of *Saussurea* occurred in the Miocene (Figure 3), which was consistent with our parallel study using whole plastome sequences (ca. 8.38–15.35 Mya) [33] and the result based on the nuclear internal transcribed spacer (ITS) dataset (ca. 11.7–14.4 Mya) [30]. A much older divergence time was provided by the study of Xu et al. [32] (ca.17.1 Mya) using plastid protein-coding genes. Additionally, the estimation of divergence time between *Saussurea* and *Cynara* (ca. 22.3 Mya) was younger than the study of Barres et al. [34] (ca. 28.3 Mya), which may be attributed to the sparse sampling of related genera of *Saussurea* in the present study. Nonetheless, all these studies suggest that the rapid diversification of *Saussurea* was related to the uplift of the QTP and adjacent mountains (e.g., Hengduan Mountains). Overall, our results support a Miocene diversification of *Saussurea* triggered by extensive orogenic movements of the QTP and subsequently mediated by genomic evolution to adapt to the fluctuant environments. 

Orthologous cluster analysis of *Saussurea* transcriptomes detected some important gene families specific to the alpine species (Figure 4). Subsequent functional characterization provided genetic evidence of high-altitude adaptation. For example, some gene families specific to alpine species are related to oxidation-redox reactions (oxidoreductase activity; GO: 0016705, GO:0016491), which is one of the most important types of reactions in the biological process. Alpine species may recruit more members involving in the oxidoreductase activity to strengthen the adaptive capability under a hypoxic environment. In high-altitude environments, cold acclimation is vital for the survival of species. A key function of cold acclimation is to stabilize membranes against freezing injury [35]. Multiple mechanisms have been documented to be involved in this stabilization, such as changes in lipid composition and accumulation of sugar content [35,36,37]. Some biological processes we detected specific to alpine species, such as the pectin catabolic process (GO:0045490), lipid transport (GO:0006869), and polysaccharide metabolic process (GO:0005976), may protect the plant from cold damage by regulating the physical state of the plasma membrane. Moreover, numerous PSGs functionally enriched in the stabilization of membrane potential (GO:0030322), response to freezing (GO:0050826), and response to temperature stimulus (GO:0009266) pathways may help plants avoid freezing injury from cold climate by regulating cell membrane permeability.

Positive selection analysis in phylogenetic context with the branch model showed most of the genes displaying a d*N*/d*S* ratio <1, indicating that most genes are undergoing purifying selection. This is true for species inhabiting all altitude distributions. Functional annotation revealed that those PSGs are closely related to the abiotic stresses of high-altitude environments produced by strong UV radiation, hypoxia, or fluctuate temperatures. The highly energetic UV radiation in the QTP is harmful to all living species since it causes direct damage to the DNA, RNA, and proteins of organisms. Some PSGs were annotated to homolog proteins of *A. thaliana* that are involved in DNA repair and response to UV-B radiation, such as RAD54, a DNA repair/recombination protein essential in homologous recombination for repairing DNA double-strand breaks [38], RUP2 involved in balancing UV-B-specific responses and ensuring normal plant growth [39], and TT5, a chalcone-flavonone isomerase family protein involved in flavonoid biosynthesis. Flavonoids function as antioxidants that reduce DNA damage caused by reactive oxygen species (ROS) accumulation, which is induced by abiotic stresses such as extreme temperatures, UV radiation, and drought, and thus is crucial for species adapting to extreme environments [40]. These results were consistent with previous studies of high-altitude adaptation [6,22,41], suggesting that DNA-repair and UV-B-response related PSGs play vital roles for alpine species to survive in harsh environments.

Furthermore, extreme environmental conditions may impact several key biological processes including nutrient or energy intake, photosynthesis, and reproductive process [41]. Our functional annotation showed many PSGs involved in the nitrogen utilization, reproductive process, growth, and metabolic process (Supplementary Table S3). PSGs participating in metabolic processes of important compounds, such as glutamate and sulfur, were annotated in GO biological processes and KEGG pathways. Glutamate plays a central role in plant nitrogen metabolism and is crucial for many metabolic functions, including protein and glutathione synthesis, energy production, maintenance of optimal antioxidant status, and immune function [42]. Nitrogen utilization is vital for alpine species of *Saussurea* because nutrient deficiency, especially nitrogen deficiency is a typical feature of the alpine screes habitats [43,44]. The uptake and assimilation of sulfur and nitrogen are strongly related, which is an important issue in plant nutrition [45]. Moreover, sulfur plays an important role in fundamental processes such as electron transport, structure, and regulation, and is also associated with photosynthetic oxygen production, abiotic and biotic stress resistance, and secondary metabolism [45,46]. Reproductive success is critical to the continuation of species. Genes involved in the meiotic process (e.g., GO:0051754, GO:0070601, GO:0033046), recognition of pollen (GO:0048544), floral organ formation (GO:0048449), and seed dormancy process (GO:0010162, GO:0022611) were detected under positive selection. These genes provide evidence that alpine species of *Saussurea* have evolved specific reproductive strategies via positively selected genes associated with the meiotic, flowering, and seed dormancy process for adapting to the short growing season in the harsh environments of the QTP.

## 4. Materials and Methods 

### 4.1. Sample Collection and Transcriptome Sequencing 

Five *Saussurea* species were sampled for RNA-seq, of which three (*S. pachyneura, S. salwinensis,* and *S. velutina*) were collected from the QTP alpine zone of Baima Snow Mountain, De Qin County, Yunnan Province, China (between 4550 and 4620 m), and the other two (*S. amurensis* and *S. amara*) were collected from the Changbai Mountain, Jilin Province, China (between 150 and 350 m) (Supplementary Table S1). To obtain a sufficient level of transcripts, multiple tissues, including stems, fresh flowers, and leaves, were collected from living plants, mixed and stored in liquid nitrogen. Total RNA was extracted using the TRIzol^®^ Reagent (Invitrogen, Shanghai, China). The concentration of the total RNA was detected by Nanodrop 2000, and the quality was checked using 2% agarose gel electrophoresis. For each species, a paired-end cDNA library was constructed using TruSeq Stranded mRNA Library Prep Kit (Illumina), as described in the Illumina manual, and was sequenced using the HiSeq 2000 sequencing platform. 

### 4.2. Transcriptome Assembly and Annotation

Raw RNA-seq reads were cleaned for quality using Cutadapt v.1.16 [47] to remove potential adapter sequences and low-quality regions with options “-q 20 -m 20”. The clean reads were checked with FastQC v.0.11.4 (https://www.bioinformatics.babraham.ac.uk/projects/fastqc/, accessed on 5 June 2021) and were *de novo* assembled using Trinity v.2.6.6 [48] with default parameters. Redundant contigs were eliminated for the raw assemblies (transcripts) using CD-Hit v.4.6 [49] to obtain non-redundant transcripts (hereafter unigenes). TransRate v.1.0.3 [50] was used to calculate the statistics for evaluating the *de novo* transcriptome assemblies. BUSCO v.4.1.4 [51] was used to assess the completeness of the assemblies by employing the embryophyta_odb10 database. TransDecoder v.5.5 [13] was utilized to predict coding regions of all unigenes and translate them into protein sequences with a minimum open reading frame (ORF) length of 100 amino acids. For functional annotation, all unigenes were annotated based on similarity to the public NCBI non-redundant protein database (NR), Swiss-Prot protein database (Swiss-Prot, http://www.expasy.ch/sprot, accessed on 5 June 2021), Pfam (https://pfam.xfam.org/, accessed on 5 June 2021), Cluster of Orthologous Groups database (COG, http://www.ncbi.nlm.nih.gov/COG/, accessed on 5 June 2021) and Kyoto Encyclopedia of Genes and Genomes (KEGG, http://www.genome.jp/kegg/, accessed on 5 June 2021) using BLASTP v.2.2.25 [52] with an e-value cut-off of 1 × 10^−5^. The NR annotation results were used to obtain the Gene Ontology (GO) term assignments of the unigenes with the BLAST2GO v.3.1 [53] (with e-value < 1 × 10^−5^) program.

### 4.3. Estimation of Divergence Time 

To estimate the divergence time of *Saussurea* species, the amino acid sequences of seven Asteraceae species (*Artemisia annua, Carthamus tinctorius, Chrysanthemum nankingense, Cynara cardunculus, Helianthus annuus, Lactuca sativa, Mikania micrantha*) and other two outgroups (*Coffea canephora, Solanum lycopersicum*) were downloaded from the National Center for Biotechnology Information (NCBI) and Phytozome (version 12.1; https://phytozome.jgi.doe.gov/, accessed on 5 June 2021). Orthologous groups of these species were determined by OrthoFinder v.2.4.1 [54]. Amino acid sequences of single-copy orthologous genes were aligned using MAFFT v.7.470 [55]. Ambiguous positions of the alignments were deleted in TrimAL v.1.2 [56] with the “-automated1” command. The sequence alignments were then concatenated and were used to construct a maximum likelihood (ML) phylogenetic tree using IQ-TREE v.1.6.12 [57] with ModelFinder [58] determining the best-fit substitution model. Divergence time was estimated in the MCMCtree program of PAML v.4.9 [59] using the approximate likelihood method with an independent substitution rate (clock = 2) and the birth-death prior. The ML tree and the concatenated translated nucleotide alignments of 1136 single-copy orthologs (see Results) were used as input of MCMCtree. In our analysis, the MCMCtree discarded the first 1000,000 iterations as burn-in, and then it sampled every 50 iterations until it gathered 200,000 samples. In total, the MCMCtree ran 1000,000 + 50 × 200,000 = 11,000,000 iterations. Two runs were conducted for checking convergence, and Tracer v.1.7.1 [60] was to assess the effective sample size (ESS > 200) of each parameter. The divergence time between Asteraceae and outgroups was calibrated to 93–107 Mya based on the TimeTree database (http://www.timetree.org/, accessed on 5 June 2021) [61].

### 4.4. Evolutionary Analysis 

OrthoVenn2 web platform [62] was used to perform comparison and annotation of orthologous gene clusters among five *Saussurea* species, with the following parameters: e-value < 1 × 10^−5^ and an inflation value 1.5. A Venn diagram was generated to show the distribution of shared gene families among selected species. The lineage-specific gene families of three alpine species (*S. pachyneura, S. salwinensis,* and *S. velutina*) were annotated using the non-redundant protein database in UniProt and the corresponding GO enrichment was performed in OrthoVenn2. 

### 4.5. Phylogenetic Tests of Positive Selection

To detect signs of positive selection on protein-coding genes along specific lineage, we used the branch model [63] in the codeml program of PAML [58] to estimate the ratio (ω) of the nonsynonymous substitution rate (d*N*) to the synonymous substitutions rate (d*S*). We used orthologous groups of five *Saussurea* species as input. Three analyses were conducted, in which a foreground branch was specified as the clade of *S. pachyneura, S. salwinensis,* and *S. velutina*, separately. We deleted all gaps (clean data = 1) from the alignments to lower the effect of ambiguous bases on the inference of positive selection. Likelihood ratio tests were used to determine whether positive selection occurred on the foreground branch. A gene with a *p*-value <0.05 was considered as a positively selected gene (PSG). Protein sequences of PSGs were then annotated to the proteome of *A. thaliana* downloaded from the Arabidopsis Information Resource (TAIR11) database using BLASTP with an e-value cut-off of 1 × 10^−5^ and *-max_target_seqs* of 5. Functional enrichment analysis was conducted in Metascape web-based portal [63]. Pathway terms with a *p*-value <0.01 and an enrichment factor >1.5 (the enrichment factor is the ratio between the observed counts and the counts expected by chance) were collected and grouped into clusters based on their membership similarities [64].

## 5. Conclusions

Despite a fundamental understanding of the taxonomy, phylogeny, and ecology of *Saussurea* [25,30,31,32], our knowledge about the genetic mechanism behind adaptation to high-altitude habitats is still lacking. Transcriptomic data generated by the present study provided a good chance to investigate the adaptive evolution of *Saussurea*. Through comparative and evolutionary genomic analyses, we identified the gene families specific to alpine species and genes showing signs of adaptive evolution. Functional annotations of these genes elucidate that they potentially possess specific traits of adaptive significance, such as membrane stabilization, response to radiation, DNA repair, and organic metabolism, demonstrating genetic signatures under the adaptation of *Saussurea* to the low temperature, high UV radiation, and hypoxia environments on the QTP. Overall, our study provided reference-level genomic resources for alpine plants while gaining important insights into how plants adapt to harsh and extreme environments. 

## Figures and Tables

**Table 1:** Summary table of assembly information for five transcriptomes of *Saussurea* species.

Species	Type	Total Sequence Number	Total Sequence Base (bp)	Percent GC (%)	Largest(bp)	Average (bp)	N50 (bp)
*S. amara*	Unigenes	92,247	70,437,963	39.69	15,736	763.58	1334
	Transcripts	185,952	179,868,433	39.95	15,736	967.28	1562
*S. amurensis*	Unigenes	147,303	84,653,535	44.78	14,904	574.69	787
	Transcripts	249,898	161,547,729	43.63	14,904	646.45	899
*S. pachyneura*	Unigenes	91,980	65,877,634	40.21	13,829	716.22	1196
	Transcripts	176,265	151,495,643	40.44	13,829	859.48	1354
*S. salwinensis*	Unigenes	120,849	87,535,736	39.30	16,755	724.34	1154
	Transcripts	270,778	237,345,971	39.83	16,755	876.53	1349
*S. velutina*	Unigenes	106,591	78,896,059	39.81	15,517	740.18	1186
	Transcripts	228,331	209,268,034	39.97	15,517	916.51	1422

**Table 2:** Summary table of annotation information for five transcriptomes of *Saussurea* species. Unigenes were annotated based on similarity to the public databases, including NCBI non-redundant protein (NR), Swiss-Prot protein (SWISS-PROT), PFAM, Cluster of Orthologous Groups (COG), Kyoto Encyclopedia of Genes and Genomes (KEGG) and Gene Ontology (GO).

Species	ORF (%)	NR (%)	SWISS-PROT (%)	PFAM (%)	COG (%)	KEGG (%)	GO (%)
*S. amara*	16,851 (18.27)	21,395 (23.19)	12,683 (13.75)	9826 (10.65)	10,440 (11.32)	8110 (8.79)	12,543 (13.60)
*S. amurensis*	28,182 (19.13)	31,705 (21.52)	22,320 (15.15)	15,886 (10.78)	21,901 (14.87)	14,179 (9.63)	22,081 (14.99)
*S. pachyneura*	15,906 (17.29)	21,642 (23.53)	12,819 (13.94)	9474 (10.30)	10,726 (11.66)	8204 (8.92)	12,674 (13.78)
*S. salwinensis*	19,979 (16.53)	26,176 (31.66)	14,722 (12.18)	11,017 (9.12)	11,890 (9.84)	9479 (7.84)	14,577 (12.06)
*S. velutina*	19,978 (18.74)	24,748 (23.22)	15,182 (14.24)	11,268 (10.57)	12,359 (11.59)	10,008 (9.39)	15,021 (14.09)

**Table 3:** GO enrichments based on the annotations in UniProt of lineage-specific gene families of three alpine *Saussurea* species.

Species	GO Term	GO Term Definition	*p*-Value
*S. pachyneura*	GO:0016705	Oxidoreductase activity, acting on paired donors, with incorporation or reduction of molecular oxygen	0.0065
	GO:0050896	Response to stimulus	0.0250
	GO:0016491	Oxidoreductase activity	0.0140
*S. salwinensis*	GO:0045490	Pectin catabolic process	0.0000
	GO:0070072	Vacuolar proton-transporting V-type ATPase complex assembly	0.0001
	GO:0005615	Extracellular space	0.0001
	GO:0009908	Flower development	0.0038
	GO:0005976	Polysaccharide metabolic process	0.0360
*S. velutina*	GO:0016787	Hydrolase activity	0.0045
	GO:0016491	Oxidoreductase activity	0.0230
	GO:0016209	Antioxidant activity	0.0210
	GO:0006725	Cellular aromatic compound metabolic process	0.0054
	GO:0050896	Response to stimulus	0.0120
	GO:0006869	Lipid transport	0.0020

## Data Availability

The raw RNA sequencing data have been deposited in the SRA under Bioproject number PRJNA566024.

## Supplementary Materials

The following are available online at https://www.mdpi.com/article/10.3390/plants10081715/s1, Figures S1–S5. GO terms of all unigenes of five *Saussurea* transcriptomes. Table S1. Sample information of five species of Saussurea. Table S2. Summary of reads information of sequenced transcriptomes. Table S3. Annotation information of positively selected genes to TAIR11 database. Table S4. Summary of functional enrichment analysis of positively selected genes.
