# Supplementary material for: Transcriptomes of Saussurea (Asteraceae) Provide Insights into High-Altitude Adaptation
Source: Plants (Basel). 2021 Aug 20;10(8):1715. doi: 10.3390/plants10081715 (PMC8402177; doi:10.3390/plants10081715)
Supplement: Supplementary file 1 [file plants-10-01715-s001.zip › Supplementary FigureS1-S5.pdf]

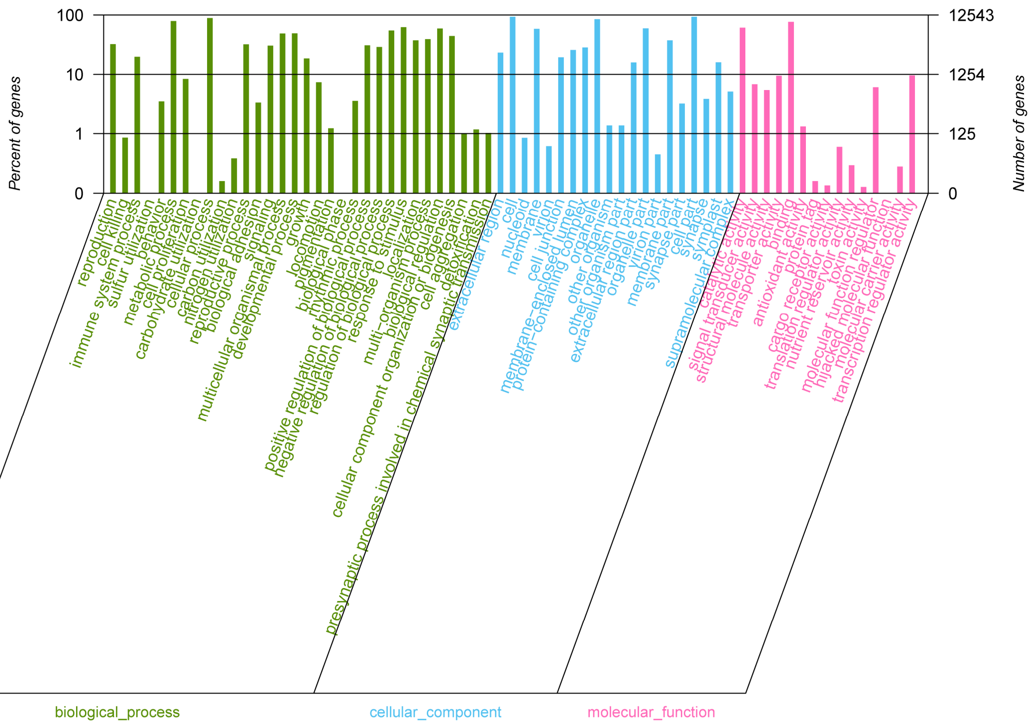

Figure S1 GO terms of all unigenes of *Saussurea amara*. Three functional categories: cellular component, molecular function and biological process are included.

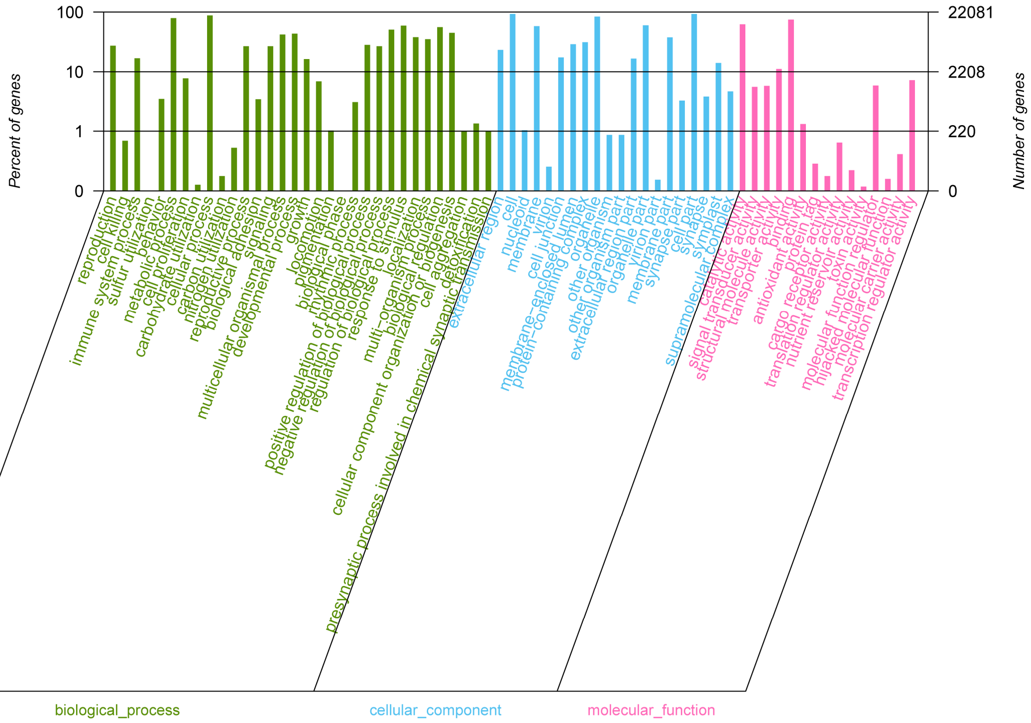

Figure S2 GO terms of all unigenes of *Saussurea amurensis*. Three functional categories: cellular component, molecular function and biological process are included.

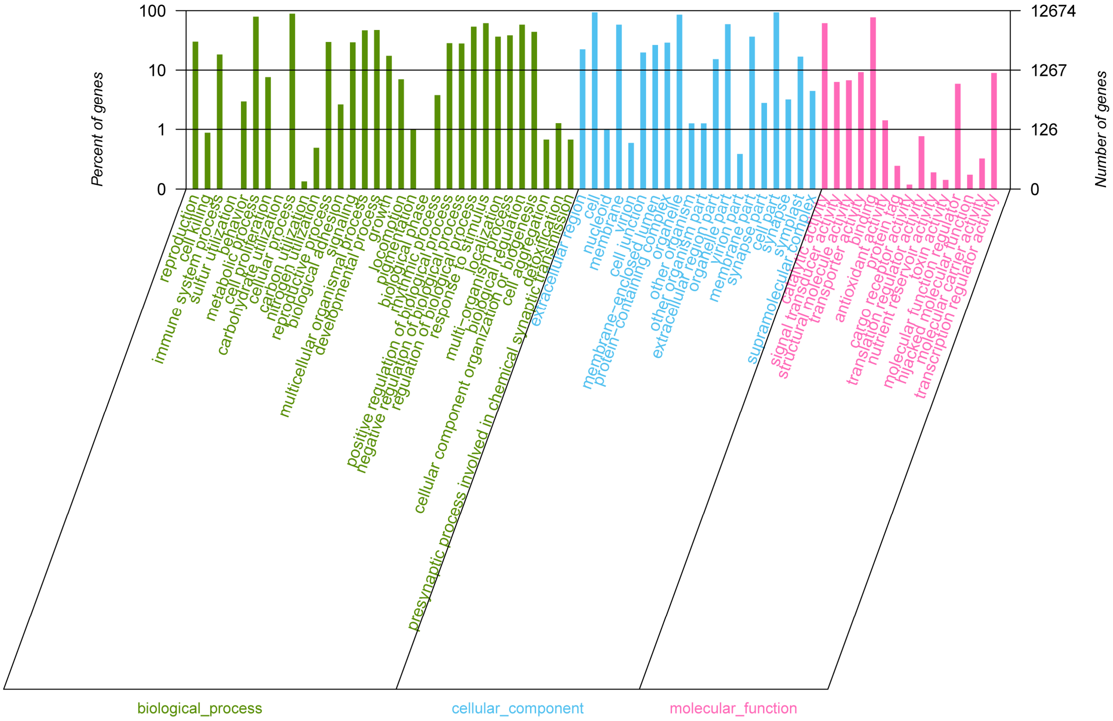

Figure S3 GO terms of all unigenes of *Saussurea pachyneura*. Three functional categories: cellular component, molecular function and biological process are included.

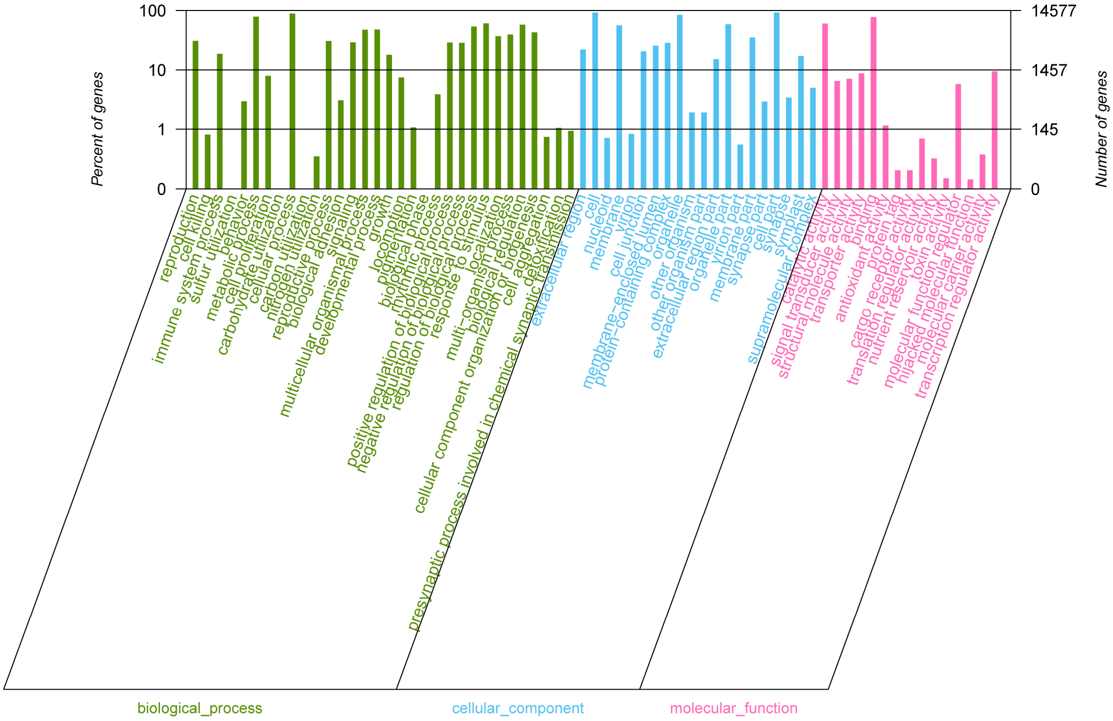

Figure S4 GO terms of all unigenes of *Saussurea salwinensis*. Three functional categories: cellular component, molecular function and biological process are included.

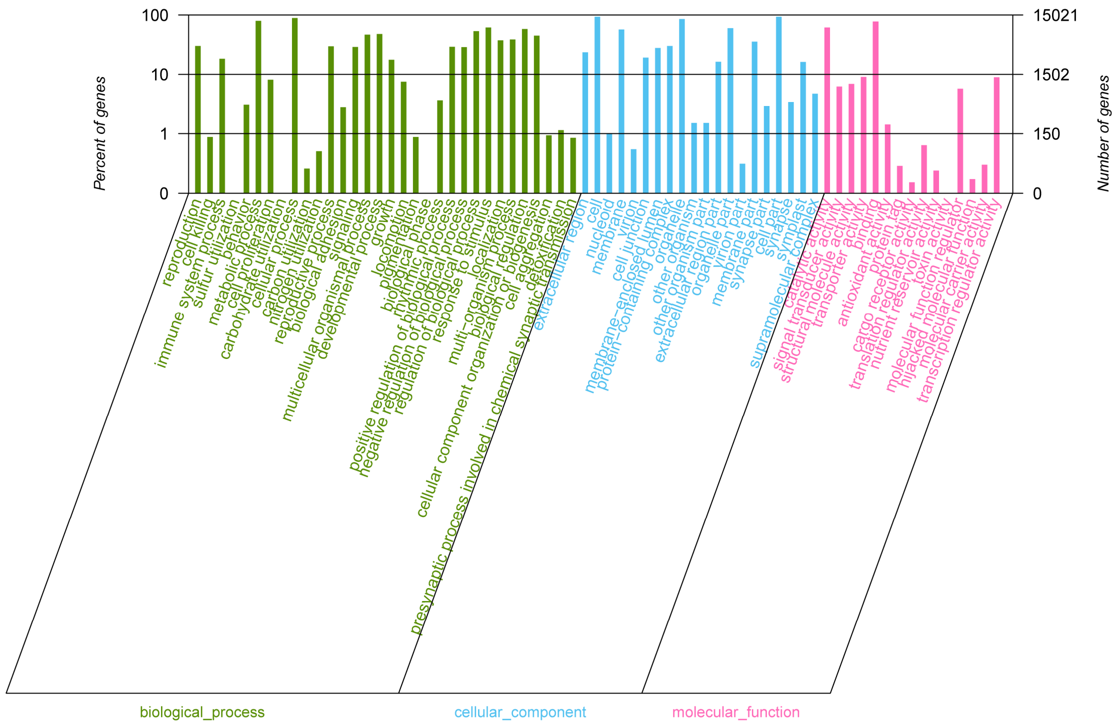

Figure S5 GO terms of all unigenes of *Saussurea veletina.* Three functional categories: cellular component, molecular function and biological process are included.
